# Involvement of NF-*κ*B/IL-6 Pathway in the Processing of Colorectal Carcinogenesis in Colitis Mice

**DOI:** 10.1155/2014/130981

**Published:** 2014-06-29

**Authors:** Hang Yang, Haili Qi, Jingli Ren, Jing Cui, Zhenfeng Li, Helge L. Waldum, Guanglin Cui

**Affiliations:** ^1^Department of Gastroenterology, The Second Affiliated Hospital of Zhengzhou University, Zhengzhou, Henan 455000, China; ^2^Department of Pathology, The Second Affiliated Hospital of Zhengzhou University, Zhengzhou, Henan 455000, China; ^3^Institute of Clinical Medicine, Norwegian University of Science and Technology, 7489 Trondheim, Norway; ^4^Institute of Health, Nord-Trøndelag University College, 7600 Levanger, Norway

## Abstract

Nuclear factor-kappaB (NF-*κ*B)/interleukin (IL-6) pathway links chronic inflammation to colitis associated cancer (CAC). In this study, we examined the dynamic temporal changes of the NF-*κ*B/IL-6 pathway during the procession of experimental CAC mouse model. Mice were sacrificed after induction for 14, 16, 18, and 22 weeks for the examination of tumor burden, inflammation degree, and protein level of NF-*κ*B and IL-6 in bowel tissues. The results showed that tumor burden and inflammation severity in the bowels were gradually increased over the observed time-points. The expressions of IL-6 and NF-*κ*B proteins were gradually increased after induction of dysplastic lesions over times. These data provide new information on the dynamic temporal changes of NF-*κ*B/IL-6 pathway in relation to CAC development that may be relevant in the design of future investigations of therapeutic interventions to effectively target CAC processes.

## 1. Introduction

Patients with ulcerative colitis (UC) are at increased risk for colorectal cancer, a phenomenon with the largest fraction being attributed to chronic inflammation [1, 2].

Growing evidence supports a role for proinflammatory mediators present in the tumor microenvironment in the pathogenesis of colitis associated colorectal cancer (CAC) [3]. Interleukin IL-6 is a pleiotropic cytokine with potent functions in promoting inflammation and neoplastic transformation and is involved in the development of CAC [4–7]. It has been found that the expression of IL-6 is increased and its expression level is correlated with the severity of disease in UC [8–10]. The promoting effect of IL-6 on the development of CAC has also been studied [3, 11–13]. Grivennikov and colleagues [12] have demonstrated that IL-6 exerts a strong promoting effect on the development of CAC in mice dosed with a low amount of chemical inflammation inducing regent dimethylhydrazine (DSS), whereas blocking of IL-6 signal by injecting IL-6 receptor antibody can successfully suppress the intestinal inflammation [14, 15] and then CAC [6, 16]. Further evidence suggests that the genes that encode IL-6 are under the control of transcriptional nuclear factor-kappa B (NF-*κ*B) [3, 17]. In Grivennikov's study [12], the authors have demonstrated that the activation of NF-*κ*B increases the production of IL-6 by lamina propria myeloid cells, while the increase of IL-6 protects the premalignant intestinal epithelial cell from apoptosis that is largely mediated by the transcription factor Stat3 [12]. Thus, it is proposed the NF-*κ*B-IL-6-Stat3 cascade is an essential stimulator for the proliferation and survival of precancerous cells in the context of chronic inflammation [12]. Interestingly, a study has shown that the DSS-evoked intestinal inflammation and CAC are significantly inhibited by the inactivation of IKK*β* (a protein kinase responsible for NF-*κ*B activation) in mice [18]. Therefore, current evidence strongly suggests that NF-*κ*B/IL-6 pathway is a key player in linking chronic inflammation to CAC [19, 20]. However, dynamic of the NF-*κ*B/IL-6 pathway during the procession of CAC has not been well characterized.

In view of these observations, we therefore performed this study to evaluate the dynamic temporal changes of NF-*κ*B/IL-6 pathway in relation to the procession of experimental CAC in mice.

## 2. Materials and Methods

### 2.1. Animals

Twenty-nine specific-pathogen-free ICR male mice aged 5 weeks (median weight of 35 g) were purchased from Shanghai Super-B&K Laboratory Animal Corp., Ltd., China, and housed in a specific-pathogen-free environment and fed with commercial pellets. All experiments were approved by the Local Animal Welfare Committee of Zhengzhou University.

CAC in mice was inducedby 1.2-dimethylhydrazine (DMH) plus dextran sulfate sodium (DSS) administration as previous reports [21, 22]. Briefly, the mice were i.p. injected with 25 mg/kg DMH (Aladdin Chemistry, Shanghai, China, MW 133.02) once weekly and kept on regular diet and water for 7 days and then received 1% DSS solution (MP Biomedicals LLC, Illkirch Cedex, France, MW 50,000) for 7 days; total 10 more DSS treatment cycles were applied until the experimental terminal (after induction for 22 weeks).

### 2.2. Tumor Number Counts and Inflammation Degree Analysis

Mice were sacrificed after induction for 14, 16, 18, and 22 weeks. Large bowels were opened longitudinally, flushed with saline, and macroscopic tumors were counted.

Then, bowel tissues were divided into two parts, one part for the protein (western blotting) study and another for the histological examination at the Department of Pathology. Embedded colorectal tissues were cut in 4 *μ*m thickened serial sections and stained with hematoxylin and eosin (H&E) staining sections. Severity of inflammation was graded as light, moderate, and high according to the inflammatory cell infiltration [23] by experienced pathologists at the Department of Pathology, the 2nd Affiliated University Hospital of Zhengzhou University, and reviewed by Senior Pathologist Dr. JR.

### 2.3. Immunohistochemistry (IHC) of IL-6 and NF-*κ*B

IHCs for IL-6 and NF-*κ*B were performed with* Vectastain Elite ABC* Kit (Vector Laboratories Inc., Burlingame, CA, USA) according to the manufacturer's instructions and our published method [24]. The following primary antibodies were used: rabbit anti-IL-6 polyclonal antibody (working dilution 1 : 100, Santa Cruz Biotechnology Inc., Santa Cruz, CA, USA) and rabbit anti-NF-*κ*B polyclonal antibody (working dilution 1 : 100, Santa Cruz Biotechnology Inc., Santa Cruz, CA, USA). Antibodies were incubated at 4°C over night. 3-Amino-9-ethylcarbazole (AEC, Vector Laboratories, Burlingame, CA, USA) was used as chromogen and slides were slightly counterstained with Mayer's hematoxylin. Negative control slides for IHCs were performed routinely: (1) primary antibodies were substituted with the isotype-matched control antibodies; (2) secondary antibody was substituted with phosphate buffered saline (PBS). Both H&E and IHC stained slides were observed under light microscopy (DM 2000, Leica Microsystems, Wetzlar, Germany).

Morphometric evaluation was performed in both the stroma and the epithelium in stained slide. In the stroma the density of IL-6 and NF-*κ*B positive cells in 3 fields with the most abundant positive cells was graded according to the method we published previously [25]. The average values per slide were used for statistical analysis.

### 2.4. Western Blotting for IL-6 and NF-*κ*B

Tissue samples were homogenized in a standard RIPA buffer, unless stated otherwise, with a cocktail of protease and phosphatase inhibitors. Cytoplasmic and nuclear proteins were prepared using NE-PER kit according to the manufacturer's instructions. Lysates were separated on a 10–15% SDS-polyacrylamide gel and proteins were transferred to PVDV membrane. Membranes were blocked in PBST buffer containing 5% skim milk for 1 hour at room temperature and probed with primary antibodies and secondary HRP-conjugated antibodies. Membranes were developed using ECL western blot detection reagent.

### 2.5. Statistical Analysis

Results were expressed as mean ± SEM (standard error of the mean) unless otherwise stated. Statistical significance was evaluated by the Mann-Whitney* U* test and the Kruskal-Wallis test. The correlation coefficients between overall bowel tumor number and the densities of IL-6 and NF-*κ*B positive cells were determined by Spearman correlation analyses. Values of *P* < 0.05 or *P* < 0.01 were considered significant.

## 3. Results

### 3.1. Tumor Incidence and Inflammation Degree in Male ICR Mice at Different Time-Points

**Figure 1:**
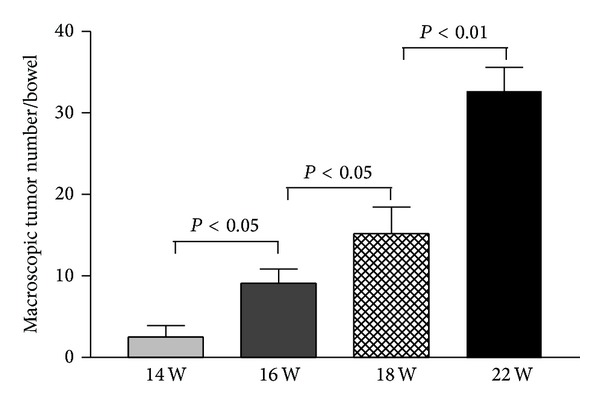
Increased tumor number in the bowels over examined time-points. The data showed that the overall tumor number in the bowels after the administration of DMH and DSS was gradually increased after induction for 14 weeks and through 22 weeks.

**Figure 2:**
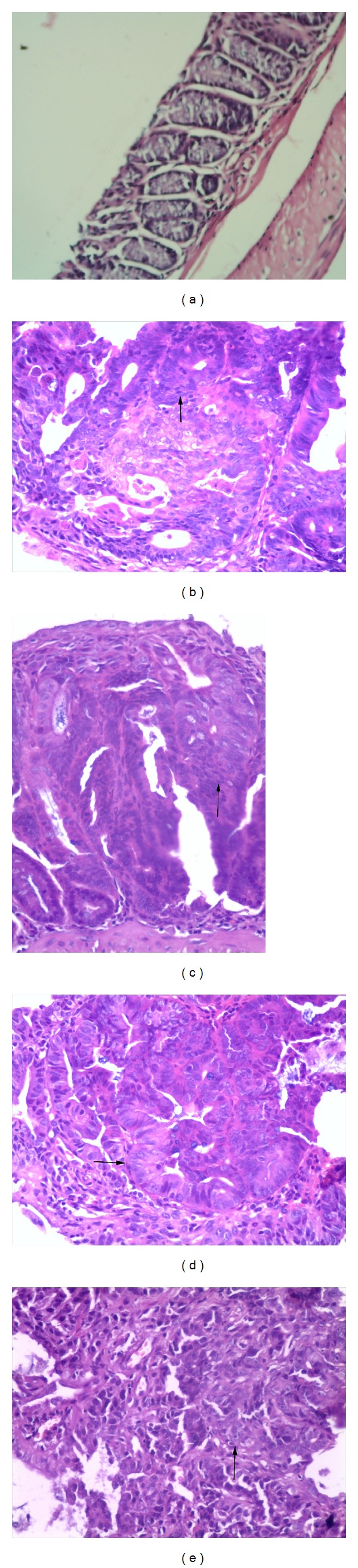
Photograph presentation of histological change of dysplasia in the bowels taken from ICR mice dosed with DMH and DSS over the examined time-points. The images from H&E stained slides showed that tumors were not observed in the controls (Figure 2(a)). However, dysplastic lesion was observed after induction for 14 weeks (arrow pointed in Figure 2(b)); the degree of dysplasia was further increased after induction for 16 weeks (arrow pointed in Figure 2(c)) and 18 weeks (arrow pointed in Figure 2(d)) and finally became cancerous lesion after the induction of 22 weeks (arrow pointed in Figure 2(e)) (Figures 1(a)–1(e): images from H&E stained slides, original magnification ×200).

Tumors were observed in the colorectal mucosa and the number of tumors was increased over observed times (Figure 1). The size of macroscopic tumors was gradually increased over times (14 weeks versus 16 weeks versus 18 weeks versus 22 weeks: 0.5 ± 0.23 mm versus 3.13 ± 1.5 mm versus 3.93 ± 1.83 mm versus 4.42 ± 1.20 mm, *P* < 0.01, the Kruskal-Wallis test). Normal histological appearance was shown in the control (Figure 2(a)); dysplastic lesion was observed in CAC mice. Dysplastic lesion in the colorectal mucosa after induction for 14 weeks was at a low grade (Figure 2(b)) and it was gradually increased over time (Figure 2(c) for the 16 weeks, Figure 2(d) for the 18 weeks) and finally became cancerous lesion after induction for 22 weeks (Figure 2(e)).

**Figure 3:**
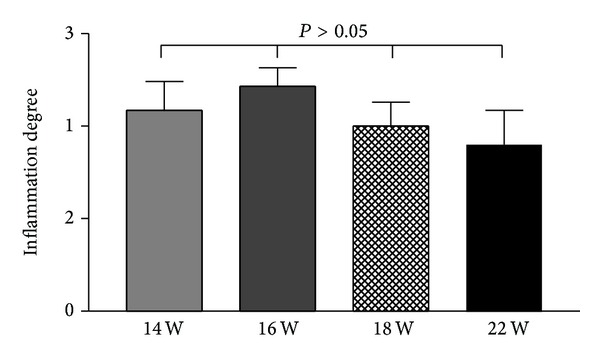
Graph analysis of bowel inflammation degree changes along the neoplastic transformation in ICR mice dosed with DMH and DSS over examined time-points. The administration of DMH and DSS for 14 weeks evoked a strong inflammation in the colorectal mucosa through 16 and 18 weeks and persisted to 22 weeks (*P* value was obtained with the Mann-Whitney* U* test).

The administration of DMH and DSS also evoked a strong inflammation in the colorectal mucosa; it was started after induction for 14 weeks and through the experimental terminal (for 22 weeks). The grading score of inflammation was increased after induction for 14 weeks and through the experimental terminal (see Figure 3).

### 3.2. The Changes of IL-6 and NF-*κ*B Positive Cell Densities in the Tumor Microenvironment during the Processing of CAC

**Figure 4:**
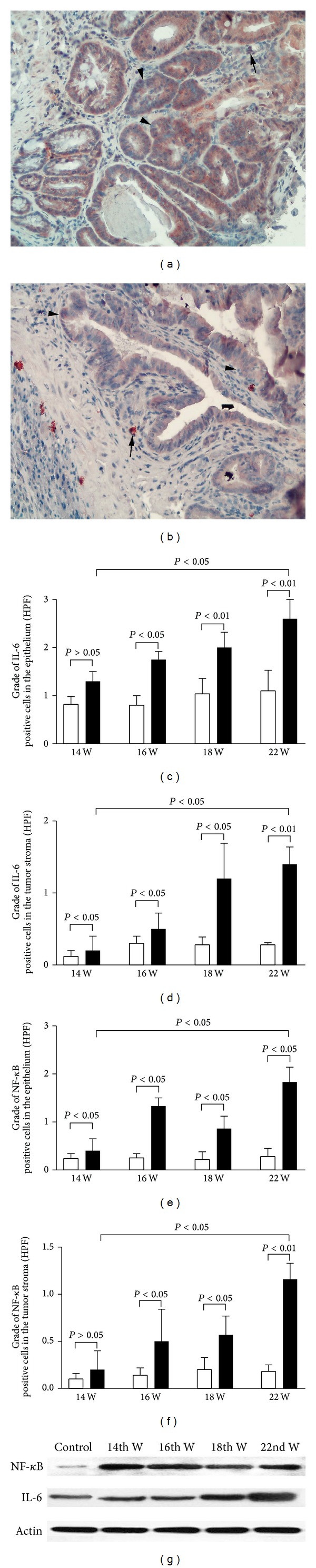
The expression patterns of IL-6 and NF-*κ*B positive cells in the tumor tissues over examined time-points. Represented immunohistochemical photos taken from CAC mice treated with DMH and DSS for 18 weeks showed that IL-6 (Figure 4(a)) and NF-*κ*B (Figure 4(b)) immunoreactivities were observed in both the transformed epithelium (arrow head pointed in Figures 4(a) and 4(b)) and tumor stroma (arrow pointed in Figures 4(a) and 4(b)). The cell density grading results confirmed the immunohistochemical observations and demonstrated that the densities of IL-6 (Figures 4(c) and 4(d)) and NF-*κ*B (Figures 4(e) and 4(f)) positive cells in both the CAC epithelium and tumor stroma were significantly increased over examined time-points (*white bar* for age matched control,* black bar* for dysplastic lesion at different examined time-points in Figures 4(c)–4(f)). The western blotting results revealed that both of the IL-6 and the NF-*κ*B proteins (Figure 4(g)) were increased in dysplastic tumor tissues after induction for 14 weeks and persisted to 22 weeks and confirmed the immunohistochemical observations as well (A&B: IHC images counterstained with hematoxylin, original magnification 200x; HPF: high power field; *P* values in Figures 4(c)–4(f) were obtained with the Mann-Whitney* U* test).

The expressions of IL-6 and NF-*κ*B in dysplastic lesion during the procession of CAC were examined with IHC over different time-points. The results showed that IL-6 immunoreactivity was observed both in the transformed epithelial cells (arrow head pointed in Figure 4(a)) and in the tumor stroma cells (arrow pointed in Figure 4(a)). After induction for 22 weeks, the CAC neoplastic epithelium was strongly positive for IL-6 (arrow head pointed in Figure 4(a)). When the density of IL-6 positive cells was graded, it was shown that the increase of IL-6 positive cell density was started after induction for 14 weeks, became even higher after induction for 16 and 18 weeks, and reached the highest level after induction for 22 weeks (experimental terminal) in both transformed epithelium (Figure 4(c)) and tumor stroma (Figure 4(d)).

The expression of NF-*κ*B in the tumor tissues was in the same change pattern as IL-6. NF-*κ*B immunoreactivity was detected both in the transformed CAC cells (arrow head pointed in Figure 4(b)) and in the tumor stroma cells (arrow pointed in Figure 4(b)). The density of NF-*κ*B positive cells was also shown in an increased trend in both transformed epithelium (Figure 4(e)) and tumor stroma (Figure 4(f)) over the observed time-points.

### 3.3. Expression of IL-6 and NF-*κ*B Proteins in the Tumor Tissues over the Observed Time-Points

The expression of IL-6 and NF-*κ*B proteins in the tumor tissues was examined with western blotting. The results confirmed the IHC observations and demonstrated increased expression of NF-*κ*B and IL-6 proteins in the tumor tissues (see Figure 4(g)) that was started after induction for 14 weeks and became more significant in the experimental terminal (after induction for 22 weeks).

### 3.4. Correlations

In order to further address the relationship between overall macroscopic tumor number in the bowel and the densities of NF-*κ*B and IL-6 in the tumor tissues, we performed correlation analyses. CAC mice from all observed time-points were included in the correlation analyses. As results present in Table 1, the analysis data showed that both of NF-*κ*B and IL-6 in the stroma and dysplastic epithelium were positively associated with the overall tumor number in the bowel (*P* < 0.01 ~ 0.05; see Table 1).

## 4. Discussion

There are growing evidences to support the view that the activation of NF-*κ*B/IL-6 pathway is involved in the development of CAC [12, 17, 18, 20, 26]. This study used an CAC mouse model to examine the dynamic temporal changes of NF-*κ*B/IL-6 pathway in relation to the procession of experimental CAC in mice. Our findings showed an elevation of NF-*κ*B/IL-6 expression that occurs over the examined time-points (after induction for 14–22 weeks) in association with the rapid increased macroscopic tumor number and dysplastic lesion degree in colitis mice. These data contribute to the growing evidence on the association between intestinal inflammation and CAC and provide new insights into the importance of NF-*κ*B/IL-6 pathway in relation to colitis associated carcinogenesis.

The link between chronic inflammation and the development/progression of human cancers has been well recognized for several decades [27–29]; recent findings support the fact that NF-*κ*B-regulated inflammatory cytokines contribute to the pathogenesis of chronic inflammation and then to the development of CRC [30, 31]. In this study, we have found that the expressions of NF-*κ*B and IL-6 were synchronously increased in the tumor tissues and accompanied with the process of CAC in mice over the examined time-points. After induction for 14 weeks, inflammation characterization was observed; the number of macroscopic tumors and the degree of dysplastic lesions in the bowel were gradually increased over the observed time-points. Furthermore, IHC analysis revealed that increased densities of IL-6 and NF-*κ*B positive cells were observed in both the transformed epithelium and the tumor stroma, which are consistent with the findings in similar CAC mouse models reported by other groups [4, 12, 18]. In this study, we have observed that the expression of both NF-*κ*B and IL-6 was in the dysplastic epithelial cells; this may suggest that NF-*κ*B expressed in the transformed cell also regulates the production of IL-6 and contributes to the elevation of NF-*κ*B and IL-6 in dysplastic lesions. Finally, our data showed a strong relationship between overall tumor number and NF-*κ*B/IL-6 positive cells at all examined time-points and indicated that NF-*κ*B/IL-6 pathway may contribute to the transformation of CAC in this colitis mouse model.

The exact mechanisms behind the protumor effect of NF-*κ*B/IL-6 in colitis associated carcinogenesis are not fully understood thus far. Many factors have been postulated to be involved in the pathogenesis of CAC [26]. IL-6, as an important proinflammatory cytokine, has been reported to be involved in both the tumor initiation and the promotion stages [12, 32]. In the initiation stage of CAC, IL-6 acts as a tumor promoter by binding to its gp130-associated receptor and activated Stat3 signal pathway [12], which is the major protumorigenic effector for IL-6. Then Stat3 will further induce the expression of genes that are related to proliferation and antiapoptosis in transformed cells [12, 20, 33]. Besides its importance during early tumor promotion, IL-6 can also accelerate tumor growth during late stages of CAC via a direct stimulation effect on proliferation and growth of tumor cells [33, 34]. In addition, IL-6 may function as an amplifier to stimulate other inflammatory cytokines. For instance, IL-6 is one of the potential stimulators for the differentiation of Th17 cell [35], while Th17 cell has been demonstrated to be important in the pathogenesis of both UC and CAC [36]. In addition, the promoting effect of IL-6 on cancer growth can also be via an activation of NF-*κ*B in the cancer cells [37]; we have shown that increased NF-*κ*B was expressed in the transformed epithelial cells and tumor stromal cells over times and suggested that the interaction between NF-*κ*B and IL-6 occurred during the procession of CAC in colitis mice. Thus, multiple mechanisms may be involved in its protumorigenic effect.

The effect of IL-6 on cellular response is through a receptor complex consisting of at least one subunit of the signal-transducing glycoprotein gp130. The activation of gp130 is classically through IL-6 binding to a membrane-bound cognate receptor (IL-6R). However, IL-6 induced biological activities are largely mediated via a natural formation of an agonistic complex with soluble IL-6 receptor (sIL-6R); this complex binds gp130 and then triggers cellular responses. This activity is termed “IL-6* trans*-signaling” and plays a critical role in promoting chronic inflammation and inflammation related cancer [33, 38, 39]. Thus, it would be interesting to further investigate whether blockade of IL-6* trans*-signaling can prevent the development of CAC in UC. Indeed, a humanized anti-IL-6 receptor monoclonal antibody (tocilizumab) that can block IL-6-mediated signal transduction by inhibiting IL-6 binding to both the transmembrane IL-6R and the sIL-6R has been developed [40]. The efficacy of tocilizumab has been confirmed in patients with chronic inflammatory diseases including Castleman disease and rheumatoid arthritis [39, 41]; it would be interesting to investigate whether tocilizumab can inhibit the development of CAC in the future.

In conclusion, NF-*κ*B/IL-6 pathway as a key protumorigenic player is dynamically increased throughout the pathological process of CAC in murine model. These findings indicate that NF-*κ*B/IL-6 pathway may contribute to all the stages of initiation, promotion, and progression of CAC in colitis mice. Future studies are necessary to fully evaluate the potential of anti-NF-*κ*B/IL-6 pathway as a preventing approach (i.e., administered at early stage before dysplastic transformation) to reduce dysplastic tumor number and as an intervention approach (i.e., administered after 14 weeks) to suppress the progression of dysplastic degree in this model.

## Figures and Tables

**Table 1:** Correlations between tumor number and densities of IL-6 and NF-*κ*B positive cells.

	Spearman *γ*	*P* value
Tumor number versus intraepithelial IL-6	0.5634	0.0078
Tumor number versus stromal IL-6	0.6359	0.0019
Tumor number versus intraepithelial NF-*κ*B	0.4466	0.0287
Tumor number versus stromal NF-*κ*B	0.5827	0.0028
